# A novel protocol for parametrization of a beam model for small stereotactic beams

**DOI:** 10.1002/acm2.70158

**Published:** 2025-07-14

**Authors:** Tim Melhus, Mohammed Ghazal, Hamza Benmakhlouf

**Affiliations:** ^1^ Department of Medical Radiation Physics and Nuclear Medicine Karolinska University Hospital Stockholm Sweden; ^2^ Department of Oncology‐Pathology Karolinska Institute Stockholm Sweden

**Keywords:** beam‐model optimization, dosimetric leaf gap, effective spot size, linac stereotactic radiotherapy

## Abstract

**Introduction:**

Most Treatment Planning Systems (TPS) utilize parameterized multi‐source models to represent the radiation beam of linacs. However, small stereotactic beams require special attention due to the importance of correct spot‐ and field‐size definition. The purpose of this work is to develop a protocol for configuring a beam model that provides accurate representation of stereotactic beams.

**Material and methods:**

6 MV flattening filter free (FFF) dose‐profiles were measured for Millennium multileaf collimator (MLC)‐collimated field sizes (2 × 2 cm^2^–5 × 5 cm^2^). They served as a reference against which corresponding TPS‐profiles were optimized by configuring the Dosimetric Leaf Gap (DLG) and the Effective Spot Size (ESS). The optimized model was evaluated by comparing calculated and measured stereotactic radiotherapy (SRT)‐plans. The proposed protocol was implemented on Anisotropic Analytical Algorithm (AAA) and Acuros XB in the Eclipse TPS.

**Results:**

Improvement in the agreement between measured and modelled profiles are identified using the suggested protocol. The DLG was determined at an optimum of 0.9 mm for both models whereas the ESS was determined to be (0.5, 0) mm and (1, 0,5) mm for AAA and Acuros XB, respectively. Compared to the standardly configured clinical model, the optimized AAA (Acuros XB) model showed an average improvement in gamma pass‐rates of 2% (3%) with film measurements.

**Conclusions:**

The standard protocol for TPS commissioning was shown to produce a sub‐optimal beam model for small treatment volumes. The proposed protocol in this work results in a better modelling of small stereotactic beams.

## 1 INTRODUCTION

In recent years, commercial treatment planning systems (TPSs) have replaced the correction‐based dose calculation models with more complex algorithms such as Monte Carlo, linear Boltzmann transport equation solvers, and semi‐analytical convolution/superposition models.^1^ While the introduction and clinical implementation of these algorithms have improved the dose calculation accuracy considerably,^2^, ^3^ they are all subject to the inherent approximations employed when constructing the algorithms as well as the TPS's modelling of energy fluence and any approximations made therein. The aforementioned dose calculation methods all require detailed characterization of the radiation source in order to achieve satisfactory accuracy,^4^ a process which is often established using a parameterized multi‐source model.^5^, ^6^ The parameters required by a TPS in order to configure a beam model of any given linear accelerator (linac) are typically derived from a set of percentage depth dose‐, lateral dose profile‐ and output factor measurements acquired during beam commissioing,^6^, ^7^, ^8^ accounting for the full range of field sizes achievable by the linac. Differences in the physical and modelled beam characteristics between different linacs, together with approximations and simplifications in the algorithms implemented within the TPS, furthermore necessitate parameters that the user can configure to adapt the beam model to individual linacs.^8^ The optimization of these user‐configurable parameters is crucial for dosimetric accuracy, especially for small radiation fields where the loss of lateral charged particle equilibrium among other small field effects may compromise the calculation accuracy.^9^, ^10^, ^11^

In the case of commercially available TPSs, the vendor provides the user with measurement protocols and internally developed recommendations on how to optimize these user‐configurable parameters.^12^, ^13^, ^14^ Several authors, however, have found large discrepancies between measured and modelled dose distributions when the vendor‐proposed protocols have been used to optimize the beam model parameters, most notably in cases where the treatment type and target volumes have not been adequately represented during the parameter optimization, and have therefore proposed alternative protocols for the optimization procedure.^13^, ^14^, ^15^, ^16^, ^17^, ^18^, ^19^ Together with an increasing demand for dosimetric accuracy to small target volumes, this raises the question of whether the vendor‐proposed optimization protocols can provide the user with a sufficiently accurate estimation of the dose, or if a separate beam model intended only for stereotactic radiotherapy (SRT) planning should be configured at each center.

The purpose of this work is to develop a protocol with which the beam model parameters are explicitly configured to match measurements of SRT fields. The protocol is specifically aimed at yielding a beam model which improves the calculations of small field dose distributions delivered using the dynamic conformal arc therapy (DCAT) technique. The impact of the developed protocol on the dose calculation accuracy is evaluated by comparing calculated and measured dose distributions using gamma‐evaluation,^20^ and by comparing the resulting model against a clinically commissioned beam model. The SRT‐optimized dose calculation models are also investigated in terms of dosimetric accuracy for larger treatment volumes as well as plans delivered using the volumetrically modulated arc therapy (VMAT) technique.

## 2 MATERIALS AND METHODS

The presented work was conducted on a 6 MV flattening filter free (FFF) photon beam generated by a Varian TrueBeam linac equipped with the 120 Millennium multileaf collimator (MLC). The Eclipse TPS (v16.1) was used to construct a beam model of the linac, and doses were computed using both the Anisotropic Analytical Algorithm (AAA, v16.1) and Acuros XB (v16.1) algorithms at a 1 mm calculation grid size. Below is a brief description of the user‐configurable parameters investigated in this work and the vendor‐suggested protocols for the optimization of the parameters. The method used to commission the clinical beam model is described, and the developed protocol for optimizing the beam model parameters specifically for SRT‐planning is detailed. In addition, the method used to evaluate the SRT‐optimized beam models against the clinical beam model is summarized.

### 2.1 The clinical beam model

In the Eclipse TPS the rounded MLC leaf‐tips are approximated as being flat‐faced for pre‐v18 algorithm versions.^12^ The discrepancy between the physical and modelled leaf‐tip geometries is intended to be corrected by the two user‐configurable parameters related to the MLC‐modelling: the MLC transmission and the dosimetric leaf gap (DLG). The MLC transmission is a single‐value parameter not taking local variations in leaf width, radiation transmission, or energy spectrum into account, representing the average radiation transmission through the MLC leaves for both leaf banks. The DLG parameter accounts for the increased transmission through the physical MLC leaf‐tips by shifting the modelled leaf‐tips and calculating doses with a leaf‐tip gap larger than the nominal gap by an amount equal to the parameter value.^12^, ^13^ Additionally, the TPS models the primary radiation source as an infinitesimal point source as opposed to having a finite extent.^12^ To model the broadening of the penumbra and take the partial source occlusion effect into account, the modelled energy fluence map is convolved using a 2D Gaussian filter at the isocenter plane.^10^, ^11^, ^12^, ^14^, ^21^ The widths of the Gaussian filter (effective spot size, ESS), represented by the standard deviation of the distribution, is user‐configurable in both the inline (ESS*_y_*) and crossline (ESS*_x_*) directions.^12^

The vendor recommends the user to configure the MLC transmission parameter by taking the ratio of measured doses in an open field to the measured dose when using the same field size with all MLC leaves closed behind the jaws, averaging the result for both leaf banks. The DLG parameter, on the other hand, is recommended to be derived from measurement results obtained by performing the sweeping gap test.^12^, ^22^ However, some authors have highlighted the need to further optimize the parameter value obtained from the sweeping gap test to achieve better agreement between measured and computed doses.^13^, ^14^, ^16^, ^17^, ^18^, ^19^ Tuning of the ESS parameters, which has a significant influence on calculated dose profiles in the penumbra region, defined as the range from 80% to 20% of the central dose, is recommended by the vendor to be performed by matching measured and calculated dose profiles.^12^

The clinically commissioned beam model was configured according to the vendor‐proposed protocols in terms of the ESS and MLC transmission parameters; The MLC transmission was taken as the ratio of doses measured in an open field to the measured dose with all MLC‐leaves closed behind the jaws averaged over both leaf banks and the ESS was configured by minimizing the discrepancy between measured and modelled jaw collimated dose profiles in both the crossline and inline directions. Likewise, the sweeping gap test was performed using an ionization chamber in a water phantom in order to measure the DLG following vendor recommendations. The DLG parameter value used to construct the clinical beam model was initially taken as the measured DLG and subsequently tweaked by comparing doses measured in a plastic phantom to modelled dose distributions in the same phantom for a few typical patient plans representative of multiple target anatomies, with the majority of plans being delivered using the VMAT technique and a field size ranging between 9 × 9 cm^2^ and 18 × 20 cm^2^. The plans were calculated for a set of parameter values and the final DLG parameter value was taken as the one which maximized the gamma pass rate (GPR) based on gamma evaluations performed using a 2% global/2 mm criterion.^13^, ^20^ For reference, the clinical beam model was configured using ESS = (ESS*_x_*, ESS*_y_*) = (0.5, 0) mm and DLG = 1.60 mm.

### 2.2 The SRT‐optimized beam models

The SRT‐optimized beam models were configured using a set of MLC‐collimated dose profile measurements acquired in both the inline and crossline directions for field sizes ranging between 2 × 2 and 5 × 5 cm^2^ in steps of 1 × 1 cm^2^, hereafter referred to as “optimization profiles”. All optimization profile measurements were acquired in a water phantom at a detector step size of 0.2–1 mm using the IBA Razor diode due to its small active volume and high spatial resolution, minimizing the volume averaging effect for small field and high dose gradient measurements.^9^, ^10^, ^11^, ^23^ The optimization profiles were acquired at a source‐to‐surface distance of 90 cm and at depths of 5, 10 and 20 cm in the crossline direction (parallel to the direction of leaf movement), and at a depth of 10 cm in the inline direction (perpendicular to the direction of leaf movement).

**FIGURE 1:**
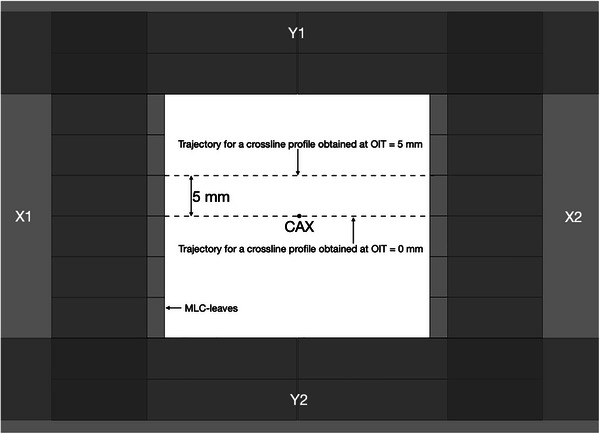
Schematic illustration of the OIT concept depicted from the beams eye‐view. The secondary collimators have been depicted as opaque to illustrate the placement of all field limiting devices, including the MLC leaf pairs defining the radiation field in the inline direction, with the X1/X2 jaw‐to‐MLC‐tip distance being set to 8 mm. Dashed lines represent the trajectories of measured profiles through the CAX and at OIT = 5 mm. CAX, central beam axis; OIT, off‐axis inline translation; MLC, millennium multileaf collimator.

The off‐axis inline translation (OIT) is a setup parameter describing the offset of profiles in the inline direction relative to the central beam axis (CAX) when measuring optimization profiles in the crossline direction (schematically illustrated in Figure 1). Due to the geometry of the MLC‐leaves and the associated local variations in radiation transmission through different parts of the leaves, it is expected that the dosimetric field size (full width at half maximum, FWHM) will vary sinusoidally over the width of a MLC‐leaf when dose profiles are measured in the crossline direction at different OITs. This is particularly apparent when using small detectors which do not average the measured signal over a large fraction of the MLC‐leaf width. The choice of measured profiles to be used for parameter optimization should thus be representative of the average dosimetric field size and penumbra width acquired over several MLC‐leaves in order to best mimic clinical SRT‐treatments, which typically extends over multiple leaf pairs. In this work, multiple crossline profiles were acquired at varying OITs ranging from−10 mm to +10 mm in steps of 0.5 mm for a 3 × 3 cm^2^ field primarily collimated by the MLC as shown in Figure 1. The most suitable OIT for the optimization profiles was subsequently determined by minimizing the difference between the penumbra width and dosimetric field size of a given measurement to the average of all measurements. For all measurements and calculations, the jaw‐to‐MLC‐tip distance was set to the same value used by the clinical beam model, 8 mm.

Multiple beam models with varying combinations of the ESS‐ and DLG parameter values were subsequently configured using both the AAA and Acuros XB algorithms. For each combination of parameter values, calculated dose profiles matching the measurement conditions (OIT, field size, direction, SSD, depth etc.) were computed and compared against the corresponding measurement, with special emphasis put on the penumbra region for two reasons: (i) our center defines the prescribed dose for SRT treatments at 67% of the CAX dose; (ii) it has been shown by other authors that changing the ESS and DLG will have a minimal impact on the calculated machine output and the plateau region of profiles for the field sizes considered in this work.^21^ The latter was also confirmed by comparing the calculated machine output from the SRT‐optimized beam models to the machine output obtained from the clinically commissioned beam model. The modelled‐ and measured dose profiles were linearly interpolated to a resolution of 0.1 mm.

The comparison of each profile‐pair (modelled and measured, for each combination of parameter values) was performed by calculating the absolute value of the difference in area under the curve of the profile‐pair in the penumbra region. This value of area between two curves served as a similarity metric and is henceforth referred to as the absolute integrated difference (AID). Minimizing this similarity metric thus yields the best agreement between the model and measurements in the penumbra region of the profiles. Since the profiles are plotted as normalized dose in unit percent as a function of off‐axis distance in unit mm, the units of AID in terms of area will be the product of these units: %mm.

The AIDs obtained using a specific set of parameter values for different field sizes were subsequently weighted according to their relative frequency in clinical treatment plans, and averaged over the depths considered in order to account for typical target volume depth variations present in most, if not all arc‐type treatments. The calculated AID for the 2 × 2 cm^2^ field size was given a relative weight of 10%, while the relative weight for the rest of the field sizes was set to 30%, reflecting the distribution of field sizes used for clinical SRT treatment plans in our center. In total, 131 and 95 different combinations of parameter values were assessed for the AAA‐ and Acuros XB beam models respectively. By constructing a three‐dimensional matrix, with the axes representing the simultaneously varying parameters (ESS*_x_*, ESS*_y_*, and DLG), and the matrix values corresponding to the field size‐weighted and depth‐averaged AID for each specific combination of parameter values, the optimal solution could be determined by identifying the minimum value within the matrix. The uncertainty of calculated AID values (presented in the Supplementary Materials) was estimated by performing reproducibility measurements of dose profiles and estimating the contributing factors from other sources of error. The combined uncertainty is found to be 2%–4%.

### 2.3 Beam model verification

To verify that the described parameter optimization protocol produces a more appropriate beam model for SRT‐planning in comparison to the clinically commissioned beam model, fifteen clinical treatment plans were measured and compared to the corresponding modelled dose distributions. While the protocol was mainly established for SRT treatments using the DCAT technique where the intensity modulation per beam is minimal, evaluating the beam model performance with the VMAT treatment technique, where the intensity modulation is generally greater than for the DCAT plans, was also of interest. All fifteen treatment plans were arbitrarily selected from a list of already clinically treated SBRT treatments which have been summarized in Table 1. The plans were all single‐isocenter plans delivered using a 15 Gy × 3 fractionation schedule to single targets located in the lung; a setup which represents 94% of clinical SBRT treatments at our institution.

**TABLE 1:** Summary of the clinical verification plans in terms of: Optimization target volume (Target volume [cm^3^], delivery technique (DCAT, or VMAT), number of arcs, total number of MU and the GPR, using 1%, 1 mm local criteria, of each model (clinical [Clin.] AAA, optimized [Opt.] AAA and optimized Acuros XB) against measured film dose.

#	Target volume (cm^3^)	Delivery technique	# Arcs	# MU	GPR Clin. AAA	GPR Opt. AAA	GPR Opt. Acuros XB
1	14.1	DCAT	1	3267	89.67	93.99	95.58
2	21.0	DCAT	1	3074	90.89	91.07	95.93
3	8.2	DCAT	1	3061	95.65	95.71	95.27
4	12.8	DCAT	1	3085	93.70	95.71	96.35
5	13.8	DCAT	1	3012	95.70	97.36	95.36
6	18.6	DCAT	1	3356	82.90	85.33	88.88
7	5.0	DCAT	1	3954	85.55	89.15	94.12
8	23.8	DCAT	1	2813	94.57	93.96	94.63
9	38.0	DCAT	1	3025	94.55	97.37	97.28
10	30.9	DCAT	1	3586	92.30	92.30	94.82
11	22.2	DCAT	1	2939	91.80	95.05	97.38
12	15.8	VMAT	2	4632	97.93	97.45	98.82
13	65.3	VMAT	2	3149	95.80	96.89	98.42
14	33.4	VMAT	2	2123	91.81	93.86	94.82
15	31.1	VMAT	2	4993	98.14	96.92	95.38

Abbreviations: AAA, anisotropic analytical algorithm; DCAT, dynamic conformal arc therapy; GPR, gamma pass rate; MU, monitor units; VMAT, volumetric modulated arc therapy.

Measurements were performed using GAFChromic EBT3 films (all from the same production lot) positioned at a depth of 5 cm in a water‐equivalent plastic phantom and with 10 cm of backscattering material. Film digitalization was done using the Epson Expression 12000XL scanner set to scan the films at a resolution of 250 dpi in transmission mode. The film orientation was the same for all measurements and scanning procedures (landscape), and the exposed area of each film was centered in the scanner for all measurements in order to minimize the lateral response artifact.^24^ The films were placed in a black envelope immediately after exposure and stored inside a dark room until at least 24 h after irradiation in order to allow the monomer polymerization to saturate before being scanned. Care was employed at all times when handling the film so as to not leave any scratches, fingerprint marks, dirt, dust or grime on the front/back polyester coating. Prior to all scans, the film and scanner windows were carefully inspected for dust, fingerprint marks, and any other interference factors, which if found would be cleaned off using a pad dampened with 85% isopropyl alcohol. Additionally, the scanner light was warmed up prior to all scans not performed within 90 s of one another in order to reduce inter‐scan variability.^24^

Film calibration was performed with a 6 MV FFF beam up to 19 Gy based on protocols outlined by AAPM TG‐235.^24^ A rational function using the net optical density as a response quantity was chosen for the calibration of the films, similar to the calibration function used by Devic et al.^25^ and measurements of treatment plans were performed using the triple‐channel dosimetry method described by Micke et al. (2011).^24^, ^26^ No lateral response artifact correction was applied due to the field sizes measured and the careful centering of the films on the scanner window.^24^ Each measurement was smoothed using an average filter with a kernel size corresponding to 1% of the measurement diagonal prior to further processing. The modelled dose distributions for each of the beam models were resampled to the same resolution as the measurement, interpolated using a linear interpolation scheme, and rigidly registered to the measurement using the correlation coefficient as the similarity metric.^27^ Considering the above‐mentioned sources of uncertainty including the uncertainty in the spatial registration between film measurements and the modelled dose distributions, the total uncertainty could be conservatively estimated as 3% (see AAPM TG‐235 and references therein^24^). The contribution of the spatial registration uncertainty on the total dosimetric uncertainty was estimated by computing the maximum distance to agreement for the 50% isodoses between all models and measurements, and multiplying the maximum distance to agreement with the average penumbra slope (in Gy/cm).

The spatially registered film measurements and modelled dose distributions for all beam models were subsequently compared using gamma‐evaluation. Film measurements was set as reference dose distributions while the modelled dose was set as the evaluated distributions. A local GPR criterion of 1% and 1 mm, was selected for the evaluation. The rationale behind selecting such a strict criterion in comparison to the uncertainty of the measurement, is to reveal the potential differences between the models, which otherwise (with a more generous criterion) would not appear.^20^ For all gamma‐evaluations a linear interpolation scheme was used to minimize the number of false positives resulting from the discrete nature of the digitalized distributions^28^, ^29^ and a low dose threshold of 10% was used for all calculations.

All calculations and processing steps were computed using an in‐house developed python script. All treatment plans had, prior to this work, been verified on the electronic portal imaging device (EPID) using the clinical beam model, with a GPR > 97% using a global 3% and 3 mm criteria. This method of clinical pre‐treatment verification was implemented in this work to verify the results of the film dosimetry by changing the passing criteria to 1%, 1 mm. The Portal Dosimetry software in Aria (Varian Medical Systems, Palo Alto, USA) was used for the evaluation.

## 3 RESULTS AND DISCUSSION

### 3.1 OIT

**FIGURE 2:**
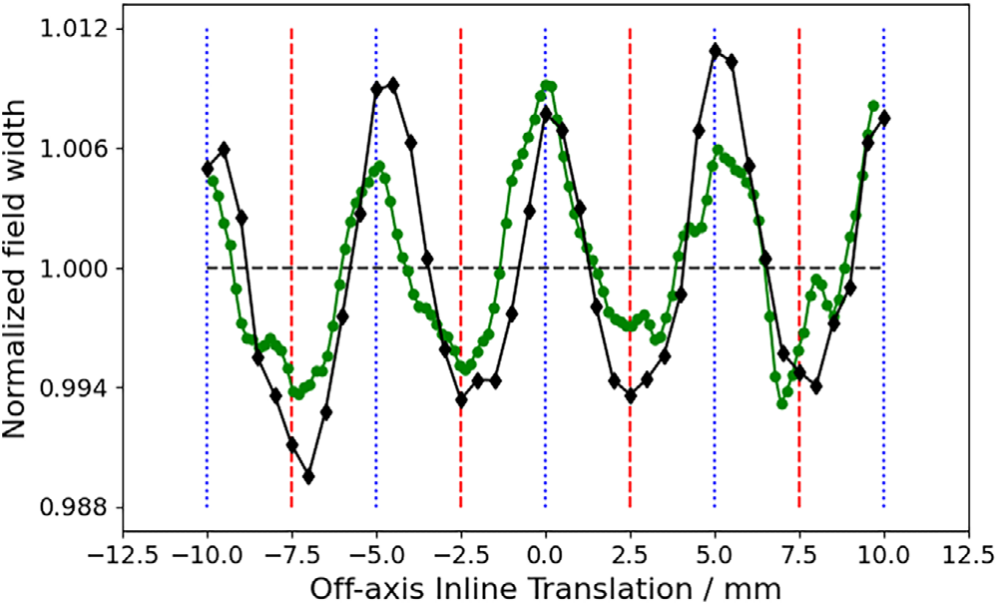
Field width (in terms of FWHM), normalized to the average, of profiles measured at MLC‐collimated 3 cm × 3 cm field size, as function of the OIT. Black diamond markers represent measurements obtained with the IBA Razor diode and the green circles were measured using Gafchromic film. Vertical blue dotted lines and red dashed lines mark the extreme position where the profile width is **expected** to be the largest and smallest, respectively. FWHM, full width half maximum; MLC, millennium multileaf collimator; OIT, off‐axis inline translation.

**FIGURE 3:**
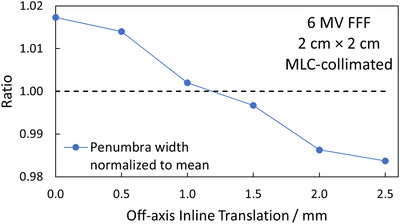
Penumbra widths, normalized to the average, of profiles measured at MLC‐collimated 2 cm × 2 cm field sizes, as function of the OIT. MLC, millennium multileaf collimator; OIT, off‐axis inline translation.

Figure 2 depicts the dosimetric field size in terms of the FWHM normalized to the average of measured 3 × 3 cm^2^ profiles as a function of OIT, obtained from measurements acquired as shown in Figure 1. As expected, the shape of the curve is sinusoidal and displays a clear tendency for the FWHM to decrease when the profile is being acquired directly under the MLC‐leaves (OIT = ± 2.5 mm, ± 7.5 mm), and increase as the OIT approaches values corresponding to profiles obtained between two MLC‐leaves (OIT = 0 mm, ± 5 mm, ± 10 mm). This trend was observed both when measuring with the IBA Razor diode, and when analyzing a GAFChromic film measurement of the same field size. The film response was quantified in terms of the optical density relative to the CAX since signal conversion into dose would not affect the apparent sinusoidal pattern of the FWHM as a function of the OIT, and the result is shown in Figure 2 as well. Similarly, Figure 3 depicts the penumbra widths obtained from measured profiles as a function of OIT for MLC‐collimated 2 × 2 cm^2^ field sizes. The increase in penumbra width with a decreasing OIT can be attributed to the increase in radiation transmission with a decreasing distance to the junction between two MLC‐leaves. Together, the figures illustrate the impact of OIT on the shape of the profiles when measurements are acquired using a small volume, high‐resolution detector, in contrast to a larger volume detector which would provide an intrinsic averaging effect over a larger fraction of the MLC‐leaf width at the cost of blurring out the dose profiles and resulting in artificially higher values for the ESS parameters.

The overall goal of these measurements was to determine an OIT which is clinically representative, and which provides a good balance between over‐ and underestimating the average profile shape over the width of several MLC‐leaves. It can be observed in Figures 2 and 3 that choosing to measure the optimization profiles in the extreme positions directly under a MLC‐leaf or directly in between leaves is not representative of the average profile over the width of a MLC‐leaf, and thus not suitable for the optimization profile measurements, neither in terms of the penumbra width nor FWHM. Under the assumption that the optimization profiles are acquired relatively close to the CAX, at an OIT where the impact of beam divergence can be considered to be negligible, the results presented in Figures 2 and 3 suggest that the best compromise, that is the most representative profile over the width of multiple MLC‐leaves, is the one measured directly in between the extreme positions (OIT = ± 1.25, ± 3.75, ± 6.25, and ± 8.75 mm), that is at one‐quarter of the leaf width. With the positioning accuracy of the measurement equipment used being 0.1 mm, an OIT of 1.2 mm was chosen as the optimal inline offset for the optimization profiles since it produces a penumbra width and FWHM close to the average penumbra width and FWHM obtained over an MLC‐leaf. It is emphasized that this optimal value of OIT only applies to the geometry of 120 Millenium MLC.

### 3.2 Beam model optimization

**FIGURE 4:**
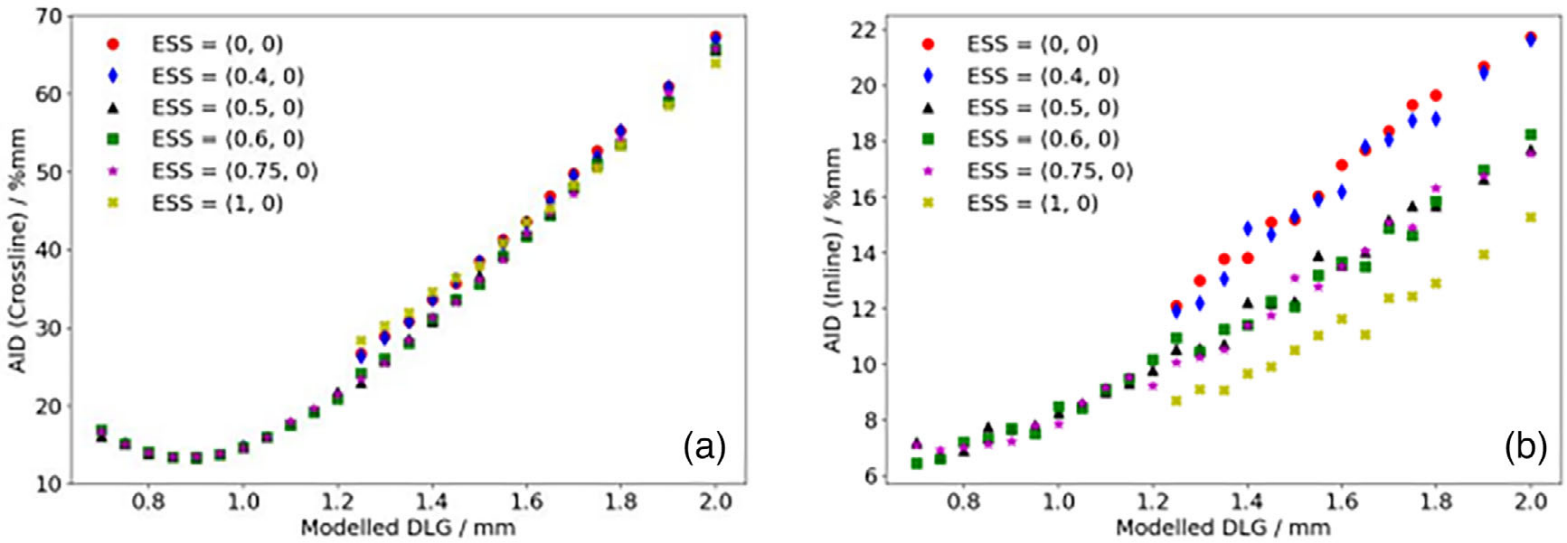
Calculated sum of AID in the penumbra region between optimization profiles measured at OIT = 1.2 mm and corresponding profiles modelled in the TPS using the AAA algorithm for a given set of beam configuration parameters in the a: crossline and b: inline directions. AAA, Anisotropic Analytical Algorithm; AID, absolute integrated difference; OIT, off‐axis inline translation; TPS, Treatment Planning Systems.

**FIGURE 5:**
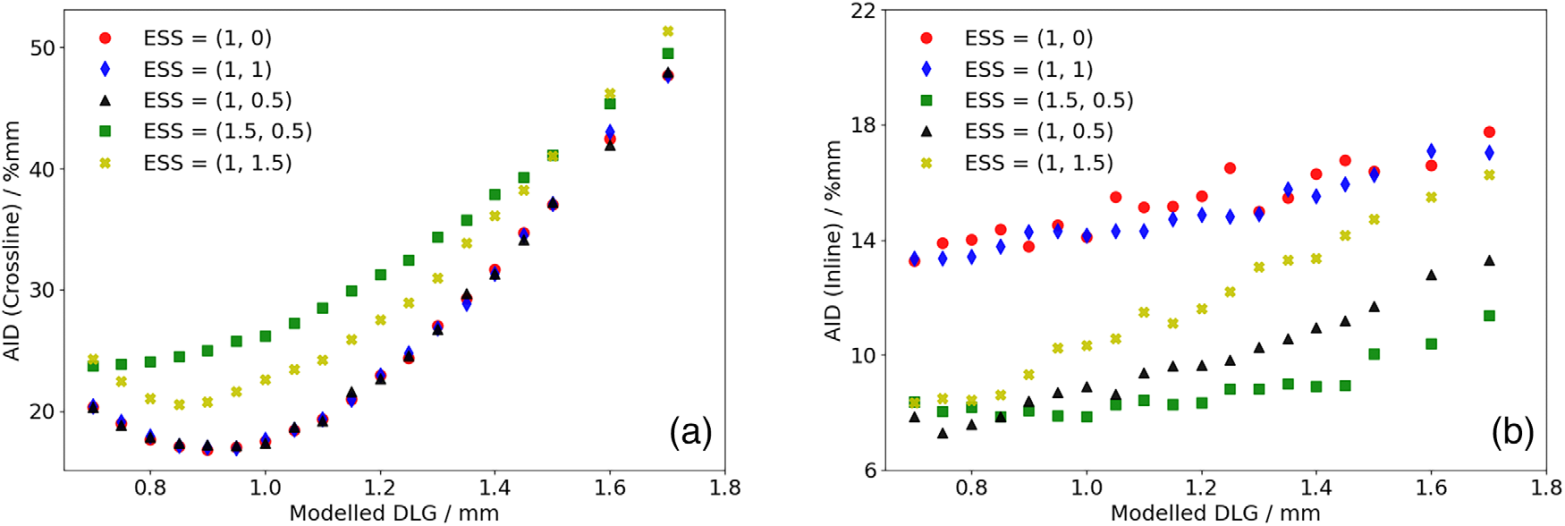
Calculated sum of AID in the penumbra region between optimization profiles measured at OIT = 1.2 mm and corresponding profiles modelled in the TPS using the Acuros XB algorithm for a given set of beam configuration parameters in the a: crossline and b: inline directions. AID, absolute integrated difference; OIT, off‐axis inline translation; TPS, Treatment Planning Systems.

Figures 4 and 5 show the calculated AID for optimization profiles measured at OIT = 1.2 mm compared to the corresponding TPS‐modelled profiles as a function of the DLG for the SRT‐optimized AAA and Acuros XB beam models, respectively. In Figure 4, representing the AID for the SRT‐optimized AAA beam model, it can be observed that the proposed protocol suggests configuring the optimal beam model with ESS (*X*, *Y*) = (0.5, 0) mm and DLG = 0.9 mm. The computed AID presented in Figure 4a for this parameter combination was found to be 0.6% and 1.3% lower than the AID corresponding to ESS = (0.6, 0) mm and (0.75, 0) mm respectively. Similarly, Figure 5 suggests that based on the proposed protocol, the optimal configuration of beam model parameters for the Acuros XB algorithm is one configured with ESS (*X*, *Y*) = (1, 0.5) mm and DLG = 0.9 mm. The SRT‐optimized beam models were thus configured using these parameter values. It should be noted from Figure 4b, representing the optimization of the SRT‐optimized AAA beam model, that the ESS in the inline direction (ESS*_y_*) is fixed to a width of 0 mm. This is motivated by observations made during the optimization procedure, where the penumbra width of the modelled profiles with ESS*_y_* = 0 mm were found to be larger than the penumbra width of the corresponding measured profiles. This means that the parameter value would need to be further decreased in order to improve the agreement between measured and modelled dose profiles, and by extension minimize the AID. Since the intention of the ESS parameter is to correct the modelled energy fluence distribution for the finite physical source size however, the parameter value has a lower bound of 0 mm, which is why ESS_y_ was fixed at this value. Unlike the SRT‐optimized AAA beam model, the Acuros XB beam model did not exhibit the same characteristics (Figure 5b), which indicates that Acuros XB is more suitable for the modelling of small field sizes, in agreement with other authors.^21^, ^30^, ^31^

It should also be noted that some beam model configurations for the SRT‐optimized AAA beam model were not investigated below a DLG parameter value of 1.25 mm, more specifically for beam configurations with ESS (*X*, *Y*) = (0, 0) mm, (0.4, 0) mm and (1, 0) mm. For these beam models, the slope of the penumbra for the modelled dose profiles did not match the slope of the penumbra for the measured dose profiles. This mismatch could not be compensated for by adjusting the DLG parameter value, resulting in the optimization constantly yielding higher AIDs. As a result, optimization efforts for these configurations were abandoned. Additionally, it should be noted that the dependence of the AID on the DLG parameter in the inline direction is simply a consequence of the positioning of the field defining MLC‐leaves illustrated in Figure 1. If the MLC‐leaves were parked at the carriages instead of at the central axis, a change in the DLG parameter value would not be expected to have any impact on the inline profiles, and by extension, the AID in the inline direction. However, having the MLC‐leaves parked at the central axis provides a simple distinction between the optimal‐ and pseudo optimal beam model parameters (Figure 5, ESS [*X*, *Y*] = [1, 0.5] mm, DLG = 0.9 mm compared to ESS [X, Y] = [1, 0] mm, DLG = 0.9 mm). It can also be clearly seen in both Figure 4b and 5b that varying the ESS parameter in the crossline direction (ESS*_x_*) while keeping the DLG at a constant parameter value impacts the calculated AID in the inline direction. This indicates that the assumed independence of ESS_x_ and ESS_y_ is not valid for the small field sizes included in this work, and that it is essential to verify both the inline and crossline directions when tuning the ESS parameter in any direction.

The DLG measured using the sweeping gap test was found to be 1.25 mm, and recall that the clinically commissioned beam model uses a DLG of 1.60 mm. These differences are significant in comparison to the accuracy of the MLC positioning.^32^, ^33^ It should be pointed out that the clinical beam model was configured according to the vendor specifications as described in the materials and methods section, with the exception of the DLG parameter value which was configured using gamma evaluation with regard to all target anatomies treated (lung, prostate, head and neck, brain, gynecological treatments etc.). The commissioning of the clinical beam model thus resulted in a DLG parameter value larger than the measured DLG, in accordance with published literature which points toward an optimal parameter value for the DLG being 0.5–0.9 mm larger than the measured DLG.^13^, ^14^, ^16^, ^18^, ^34^, ^35^ For example, Vieillevigne et al.^13^ presented an optimal DLG which was 0.7–0.8 mm larger than measured DLG, but the ESS was not considered. Other examples are Middlebrook et al.^34^ and Kim et al.^16^ who measured VMAT‐plans with no consideration of ESS and field sizes larger than those considered in this work. They reported a larger optimal DLG than the measured DLG. Also, Saez et al.^36^ proposed a procedure for optimal MLC configuration with successful outcome. However, it was applied in the RayStation TPS rendering a fair comparison with our work infeasible. In contrast, this work suggests that the optimal DLG parameter value is 0.9 mm, a parameter value 0.35 mm below the DLG measured at the linac with the sweeping gap test. This is contradictory to the findings of other authors in published literature,^13^, ^14^, ^16^, ^18^, ^34^, ^35^ which could be attributed to the differences in methodology. The presented work was uniquely focused on optimizing the parameter values to best match the penumbra regions of modelled fields to those of measured static fields, which better reflects treatment plans delivered using the DCAT technique than those delivered using the VMAT technique. The presented protocol also excludes the impact of the tongue and groove effect, which is not a user‐configurable parameter in the TPS and has been thoroughly studied and described by others.^13^, ^19^ In a report by Glenn et al.,^35^ a large variation in Eclipse DLG among multiple institutions was presented and attributed to differences in measurement equipment (i.e., the size of ionization chamber). It was further emphasized that the DLG should be critically validated by local physicist to ensure the most adequate value is chosen for local dose calculations.

**FIGURE 6:**
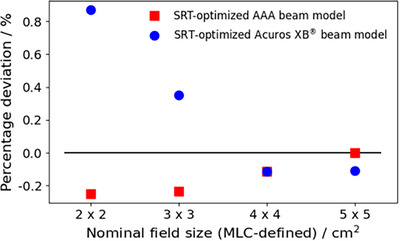
Percentage deviations in modelled output factors relative to the clinically commissioned AAA beam model for field sizes between 2 cm × 2 and 5 cm × 5 cm. Blue circles represent the deviation for the SRT‐optimized Acuros XB beam model, and red squares represent the deviation for the SRT‐optimized AAA beam model. AAA, Anisotropic Analytical Algorithm; SRT, stereotactic radiotherapy.

Figure 6 depicts the percentage deviation of calculated machine output for the SRT‐optimized beam models relative to the clinically commissioned beam model, confirming the work of Fogliata et al. who showed that the ESS and DLG parameter values have a minimal impact on the calculated machine output for fields sizes above 2 × 2 cm^2^.^21^, ^30^

### 3.3 Beam model verification

**FIGURE 7:**
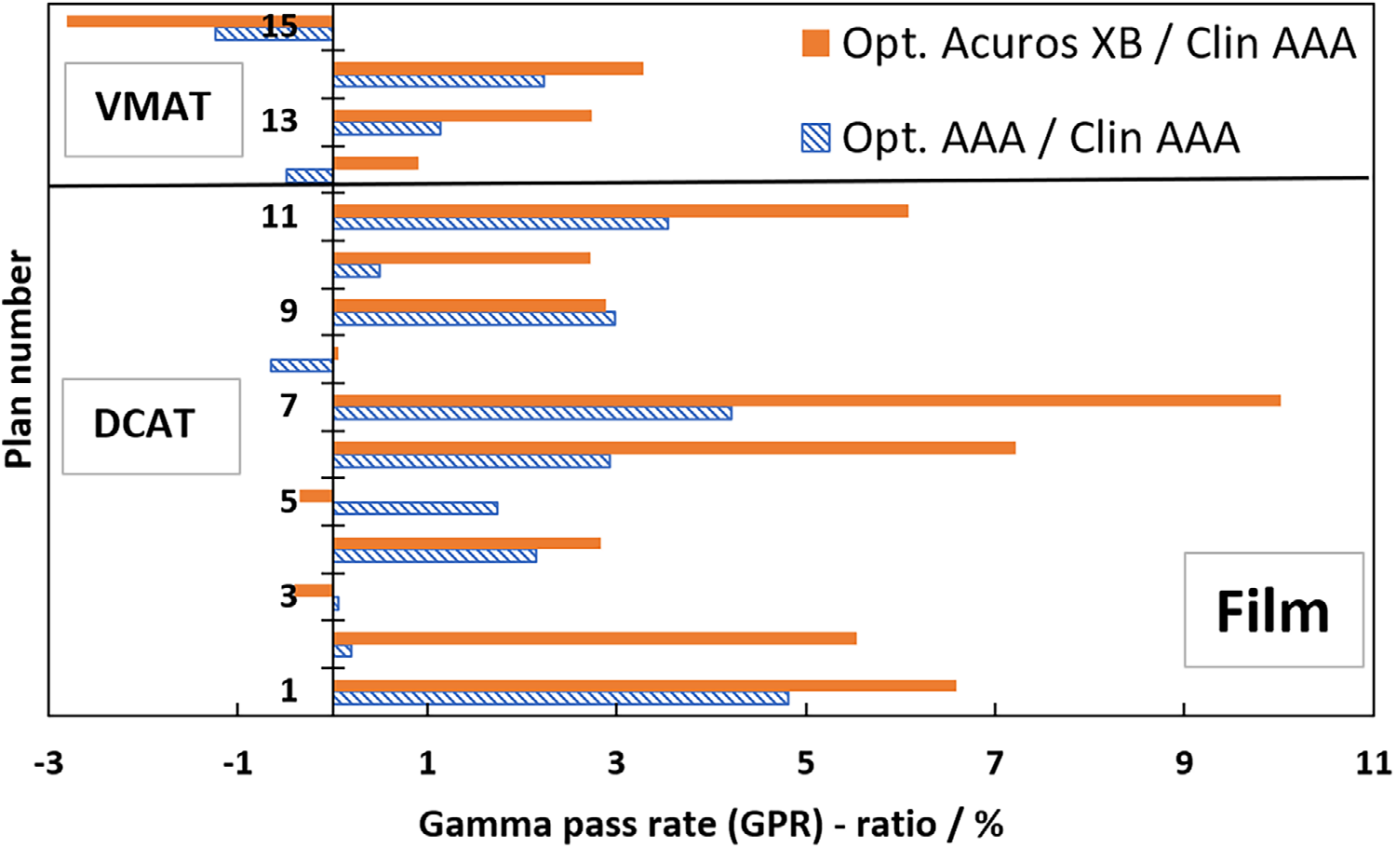
Ratios in percent of GPR of clinical treatment plans calculated with SRT‐optimized models against clinical treatment plans; dashed blue bars are SRT‐optimized AAA/clinical AAA, filled orange bars are optimized Acuros XB/clinical AAA. GPR‐analysis of modelled versus measured, all based on Gafchromic film measurements. The SRT‐optimized Acuros XB beam model is compared against the clinical AAA beam model in absence of a clinically commissioned Acuros XB beam model. AAA, Anisotropic Analytical Algorithm; GPR, gamma pass rate; SRT, stereotactic radiotherapy.

**FIGURE 8:**
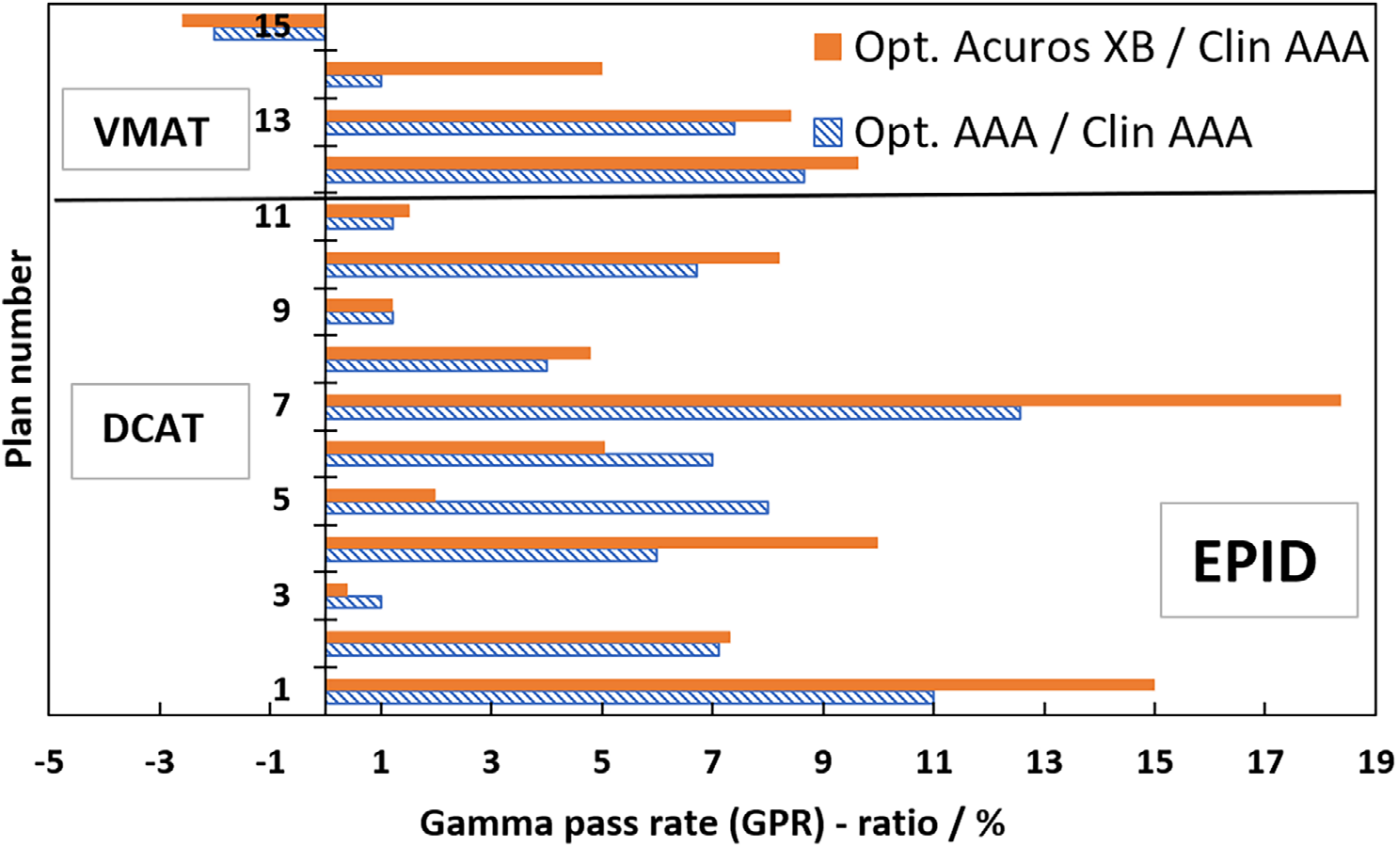
Ratios in percent of GPR of clinical treatment plans calculated with SRT‐optimized models against clinical treatment plans; dashed blue bars are SRT‐optimized AAA/clinical AAA, filled orange bars are optimized Acuros XB /clinical AAA. GPR‐analysis of modelled versus measured, all based on EPID measurements. The SRT‐optimized Acuros XB beam model is compared against the clinical AAA beam model in absence of a clinically commissioned Acuros XB beam model. AAA, Anisotropic Analytical Algorithm; EPID, Electronic Portal Imaging Device; GPR, gamma pass rate; SRT, stereotactic radiotherapy.

**FIGURE 9:**
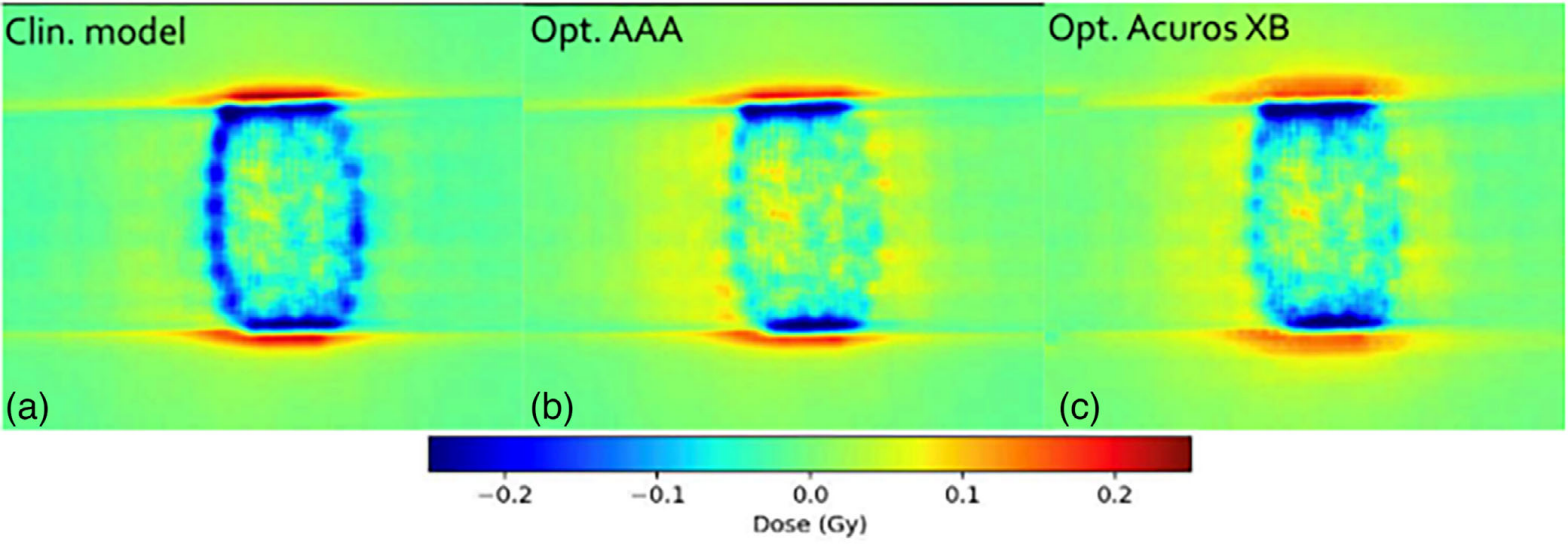
Comparison in terms of dose difference of a measured DCAT plan against: (a) clinical model, (b) optimized AAA model, and (c) optimized Acuros XB model. Measured dose was performed for plan # 4 in Table 1, using EBT3 film dosimetry. AAA, Anisotropic Analytical Algorithm; DCAT, dynamic conformal arc therapy.

**FIGURE 10:**
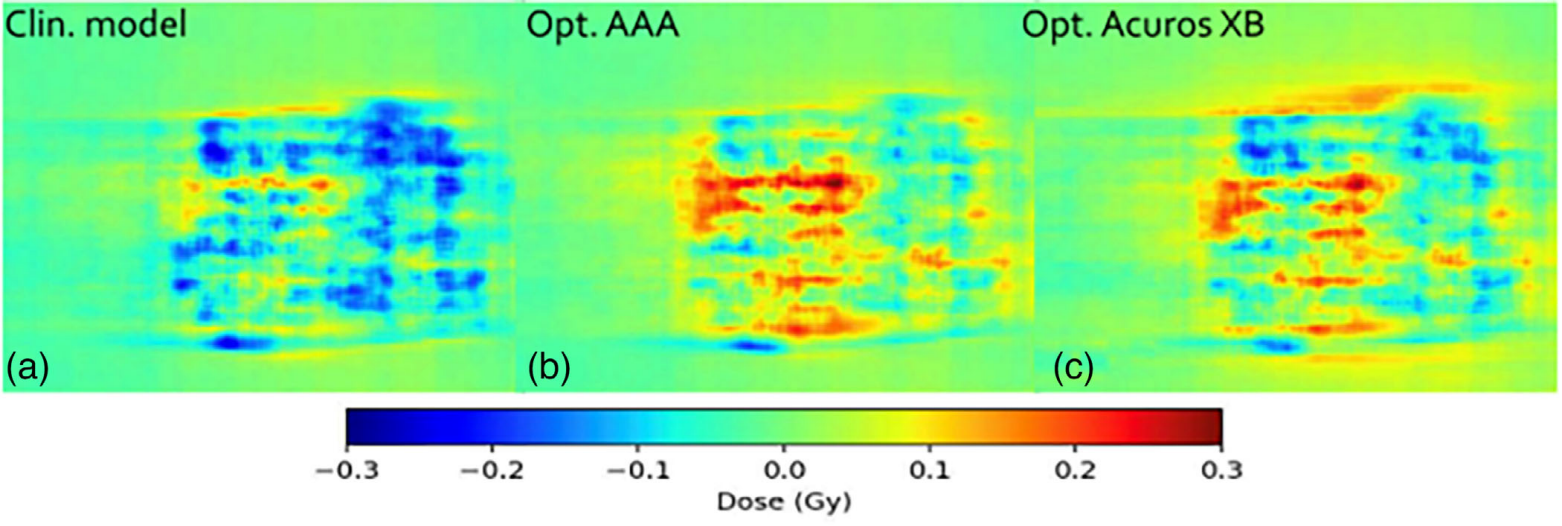
Comparison in terms of dose difference of a measured VMAT plan against: (A) clinical model, (B) optimized AAA model, and (C) optimized Acuros XB model. Measured dose was performed for plan # 13 in Table 1, using EBT3 film dosimetry. AAA, Anisotropic Analytical Algorithm; VMAT, volumetrically modulated arc therapy.

Table 1 presents the GPRs of the verification plans calculated by each model against measured film dose. Figure 7 shows the ratios of GPRs from Table 1 where the SRT‐optimized AAA‐ and Acuros XB beam models were normalized to the clinical beam model, illustrating the impact of the proposed protocol on clinical treatment plans. Corresponding results from EPID measurements are shown in Figure 8, and two illustrative examples of the comparison in terms of dose difference between measured and modelled dose distribution are shown in Figures 9 and 10.

Film measurements are considered more reliable than EPID because the dose planes for comparison were extracted directly from the clinical plans instead of the EPID‐method in which fluence maps, calculated in separate verification plans, are used for comparison. Moreover, film processing and analysis was performed using independent software while the EPID‐analysis was performed using a linac‐vendor integrated system. Therefore, henceforth all presented gamma analysis data will refer to the film measurements. Comparing measured DCAT plans against modelled plans using the SRT‐optimized AAA beam model in absolute terms, this work presents an average local GPR (1%/1 mm) of 93.4%. Similarly, the GPR for the corresponding SRT‐optimized Acuros XB beam model is 95.1%. Performing a similar analysis of the VMAT plans, GPRs of 96.3% and 96.9% were found for the SRT‐optimized AAA and Acuros XB beam models respectively. It should be noted that, for the clinical beam model, the GPR are generally higher for VMAT than DCAT. This is attributed to the fact that the clinical AAA model was configured, in terms of DLG, solely based on VMAT plans. On the other hand, for the optimized AAA and Acuros XB models, GPR for VMAT and DCAT are more similar, illustrating the value of including static field in the configuration of the beam model which represent the DCAT beam delivery. This can be observed in Figures 9 and 10. The dose difference is greatly improved in the optimized models for DCAT, especially in the penumbra region. In Figure 10, an improvement is observed but is diffused over the entire volume due to the nature of delivery technique. Generally, the results are on the same order of magnitude as published literature,^13^, ^34^, ^37^ taking into account the differences in evaluation criteria and methodology. For example, Xue et al. ^38^ showed EPID‐evaluated stereotactic VMAT plans with GPR (2%, 2 mm) between 96%–98%. Also, Vieillevigne et al. ^13^ presented an optimal DLG with an GPR agreement close to 100% with 2%, 2 mm passing criteria using the Delta4 Phantom (Scandidos, Uppsala, Sweden).

The ratios in Figure 7 are overwhelmingly positive, which means that the agreement between the measured and SRT‐optimized beam models is better than the agreement between measurements and the clinically commissioned beam model. Plan numbers 1–11 are DCAT SRT plans, and they demonstrate an average GPR improvement of 2.0% and 3.8% for the SRT‐optimized AAA‐ and Acuros XB beam models, respectively, compared to the clinically commissioned beam model. Plan numbers 12–15 are VMAT SRT plans and they indicate a low average GPR improvement over the clinical beam model of 0.4% for the SRT‐optimized AAA beam model and 1.0% for the SRT‐optimized Acuros XB beam models respectively. Verification with EPID confirms the trend of improved beam model, shown in Figure 8. As it was shown by Vieillevigne et al.,^13^ DCAT‐plans are not as sensitive as VMAT plans to variations in DLG, which is why the evaluation of this work was performed with such strict criteria. A 2%–4% improvement in accuracy of SRT plans delivered using the DCAT technique is expected. On the other hand, analysis of four VMAT SRT plans shows, on average no improvement, with individual plans being within ± 3% of the clinical beam model. Field sizes for the VMAT and DCAT clinical treatment plans are roughly the same; however, VMAT plans exhibit a higher degree of intensity modulation. For example, it can be seen in Table 1 that plan no. 15 has a higher number of MUs while delivering the same dose, meaning that the plan has a higher degree of modulation. It can also be seen in Table 1 that plan no. 15 was impacted the worse by the optimization of this work. It is simply the cost of only considering open and static fields in the parameter optimization process. As it was shown by Hernandez et al.,^19^ the higher the degree of modulation in the plans, the lower the agreement between measured and modelled dose distribution. However, no correlation was observed between the target volume and the GPR, which is most likely due to the relatively large target volumes included in this work in comparison to others who reported such a correlation, for example, Gardner et al.^39^

In conclusion, the results demonstrate a general improvement for the accuracy of DCAT SRT plans, while having only a limited impact on VMAT plans, and with the concepts of this work being applicable as long as the ESS and DLG remain the only two user‐configurable parameters. A new MLC‐model like the Enhanced Leaf Model, introduced in a newer version of the TPS (Eclipse v18) has however, been shown to provide a better representation of the treatment beam.^40^ This should not affect the demonstrated improvement based on the protocol applied in this work; however extensive studies should be conducted when commissioning the beam model for clinical use, where the presented protocol might provide a verification of an optimized beam model.

## 4 CONCLUSION

This work confirmed that the vendor‐proposed protocols for beam model commissioning yields a sub‐optimal beam model when applied to stereotactic beams. An iterative protocol was developed in this work based on configuring beam model parameters stemming from the clinical practice of specific clinics. The result demonstrates an improved representation of stereotactic treatment beams, particularly for treatment plans delivered using the DCAT technique while only minimally impacting treatment plans delivered using the VMAT technique, and the method was verified using an independent evaluation method.

## AUTHOR CONTRIBUTIONS

**Tim Melhus**: Conceptualization; Software; Methodology' Formal analysis; Investigation; Writing—Original Draft; Writing—Review & Editing; Visualization. **Mohammed Ghazal**: Conceptualization; Methodology; Investigation; Writing—Original Draft; Writing—Review & Editing; Visualization; Supervision; Final approval. **Hamza Benmakhlouf**: Conceptualization; Methodology; Writing—Review & Editing; Supervision; Project administration.

## CONFLICT OF INTEREST STATEMENT

The authors declare the following, which may be considered as potential competing interests: During the final phase of reviewing the initial manuscript, Tim Melhus was employed by RaySearch Laboratories.

## Supporting information



Supporting Information

## References

[1] De martino F , Clemente S , Graeff C , Palma G , Cella L . Dose calculation algorithms for external radiation therapy: an overview for practitioners. App Sci. 2021;11(15). doi:10.3390/app11156806

[2] Kan MW , Yu PK , Leung LH . A review on the use of grid‐based Boltzmann equation solvers for dose calculation in external photon beam treatment planning. Biomed Res Int. 2013;2013:692874. doi:10.1155/2013/692874 24066294 PMC3771252

[3] Ahnesjö A . Collapsed cone convolution of radiant energy for photon dose calculation in hetero‐geneous media. Med Phys. 1989;16(4):577‐592. doi:10.1118/1.596360 2770632

[4] Ma CMC , Chetty IJ , Deng J , et al. Beam modeling and beam model commissioning for Monte Carlo dose calculation‐based radiation therapy treatment planning: report of AAPM Task Group 157. Med Phys. 2020;47(1):e1‐e18. doi:10.1002/mp.13898 31679157

[5] Ma CM , Faddegon BA , Rogers DW , Mackie TR . Accurate characterization of Monte Carlo calculated electron beams for radiotherapy. Med Phys. 1997;24(3):401‐416. doi:10.1118/1.597908 9089592

[6] Tillikainen L , Siljamäki S , Helminen H , Alakuijala J , Pyyry J . Determination of parameters for a multiple‐source model of megavoltage photon beams using optimization methods. Phys Med Biol. 2007;52(5):1441‐1467. doi:10.1088/0031-9155/52/5/015 17301464

[7] Smilowitz JB , Das IJ , Feygelman V , et al. AAPM Medical Physics Practice Guideline 5.a.: commissioning and QA of treatment planning dose calculations—megavoltage photon and electron beams. J Appl Clin Med Phys. 2015;16(5):14‐34. doi:10.1120/jacmp.v16i5.5768 26699330 PMC5690154

[8] Ahnesjö A , Andreo P . Determination of effective bremsstrahlung spectra and electron contami‐nation for photon dose calculations. Phys Med Biol. 1989;34(10):1451‐1464. doi:10.1088/0031-9155/34/10/008 2813512

[9] Lárraga‐Gutiérrez JM , García‐Garduño OA , Herrera‐González JA , Galván de la Cruz OO . Evaluation of Acuros® XB accuracy for static small fields dose calculations based on the IAEA/AAPM TRS‐483 recommendation. Phys Med. 2021;89:140‐146. doi:10.1016/j.ejmp.2021.06.021 34365118

[10] Das IJ , Francescon P , Moran JM , et al. Report of AAPM Task Group 155: megavoltage photon beam dosimetry in small fields and non‐equilibrium conditions. Med Phys. 2021;48(10):e886‐e921. doi:10.1002/mp.15030 34101836

[11] Das IJ , Ding GX , Ahnesjö A . Small fields: nonequilibrium radiation dosimetry. Med Phys. 2008;35(1):206‐215. doi:10.1118/1.2815356 18293576

[12] Varian Medical Systems . Eclipse Photon and Electron Algorithms Reference Guide . Varian Medical Systems; 2017.

[13] Vieillevigne L , Khamphan C , Saez J , Hernandez V . On the need for tuning the dosimetric leaf gap for stereotactic treatment plans in the Eclipse treatment planning system. J Appl Clin Med Phys. 2019;20(7):68‐77. doi:10.1002/acm2.12656 PMC6612699 31225938

[14] Passal V , Barreau M , Tiplica T , Dufreneix S . Optimizing the effective spot size and the dosimetric leaf gap of the Acuros XB algorithm for VMAT treatment planning. J Appl Clin Med Phys. 2021;22(6):154‐161. doi:10.1002/acm2.13256 34042259 PMC8200512

[15] LoSasso T , Chui CS , Ling CC . Physical and dosimetric aspects of a multileaf collimation system used in the dynamic mode for implementing intensity modulated radiotherapy. Med Phys. 1998;25(10):1919‐1927. doi:10.1118/1.598381 9800699

[16] Kim J , Han JS , Hsia AT , Li S , Xu Z , Ryu S . Relationship between dosimetric leaf gap and dose calculation errors for high definition multi‐leaf collimators in radiotherapy. Phys Imaging Radiat Oncol. 2018;5:31‐36. doi:10.1016/j.phro.2018.01.003 33458366 PMC7807868

[17] Yao W , Farr JB . Determining the optimal dosimetric leaf gap setting for rounded leaf‐end multileaf collimator systems by simple test fields. J Appl Clin Med Phys. 2015;16(4):65‐77. doi:10.1120/jacmp.v16i4.5321 PMC5690020 26218999

[18] Kielar KN , Mok E , Hsu A , Wang L , Luxton G . Verification of dosimetric accuracy on the TrueBeam Six: rounded leaf effect of the high definition MLC. Med Phys. 2012;39(10):6360‐6371. doi:10.1118/1.4752444 23039672

[19] Hernandez V , Vera‐Sánchez JA , Vieillevigne L , Saez J . Commissioning of the tounge‐and‐groove modelling in treatment planning systems: from static fields to VMAT treatments. Phys Med Biol. 2017;62(16):6688‐6707. doi:10.1088/1361-6560/aa7b1a 28639942

[20] Low DA , Harms WB , Music S , Purdy JA . A technique for the quantitative evaluation of dose distributions. Med Phys. 1998;25(5):656‐661. doi:10.1118/1.598248 9608475

[21] Fogliata A , Nicolini G , Clivio A , Vanetti E , Cozzi L . Accuracy of Acuros XB and AAA dose calculation for small fields with reference to RapidArc® stereotactic treatments. Med Phys. 2011;38(11):6228‐6237. doi:10.1118/1.3654739 22047388

[22] Arnfield MR , Siebers JV , Kim JO , Wu Q , Keall PJ , Mohan R . A method for determining multileaf collimator transmission and scatter for dynamic intensity modulated radiotherapy. Med Phys. 2000;27(10):2231‐2241. doi:10.1118/1.1312190 11099190

[23] Palmans H , Andreo P , Huq MS , Seuntjens J , Christaki KE , Meghzifene A . Dosimetry of small static fields used in external photon beam radiotherapy: summary of TRS‐483, the IAEA‐AAPM International Code of Practice for reference and relative dose determination. Med Phys. 2018;45(11):e1123‐e1145. doi:10.1002/mp.13208 30247757

[24] Niroomand‐Rad A , Chiu‐Tsao ST , Grams MP , et al. Report of AAPM Task Group 235 radiochromic film dosimetry: an update to TG‐55. Med Phys. 2020;47(12):5986‐6025. doi:10.1002/mp.14497 32990328

[25] Devic S , Tomic N , Lewis D . Reference radiochromic film dosimetry: review of technical aspects. Phys Med. 2016;32(4):541‐556. doi:10.1016/j.ejmp.2016.02.008 27020097

[26] Micke A , Lewis DF , Yu X . Multichannel film dosimetry with nonuniformity correction. Med Phys. 2011;38(5):2523‐2534. doi:10.1118/1.3576105 21776787

[27] Yoo TS , Ackerman MJ , Lorensen WE , et al. Engineering and algorithm design for an image processing api: a technical report on ITK–the Insight Toolkit. Stud Health Technol Inform. 2002;85:586‐592. 15458157

[28] Ju T , Simpson T , Deasy JO , Low DA . Geometric interpretation of the gamma dose distribution comparison technique: interpolation‐free calculation. Med Phys. 2008;35(3):879‐887. doi:10.1118/1.2836952 18404924

[29] Depuydt T , Van Esch A , Huyskens DP . A quantitative evaluation of IMRT dose distributions: refinement and clinical assessment of the gamma evaluation. Radiother Oncol. 2002;62(3):309‐319. doi:10.1016/s0167-8140(01)00497-2 12175562

[30] Fogliata A , Lobefalo F , Reggiori G , et al. Evaluation of the dose calculation accuracy for small fields defined by jaw or MLC for AAA and Acuros XB algorithms. Med Phys. 2016;43(10):5685. doi:10.1118/1.4963219 27782735

[31] Younes T , Chatrie F , Zinutti M , Simon L , Fares G , Vieillevigne L . Optimization of the Eclipse TPS beam configuration parameters for small field dosimetry using Monte Carlo simulations and experimental measurements. Phys Med. 2023;114:103141. 37820506 10.1016/j.ejmp.2023.103141

[32] Enomoto H , Fujita Y , Matsumoto S , et al. Dosimetric impact of MLC positional errors on dose distribution in IMRT. J Appl Clin Med Phys. 2024;25:e14158. doi:10.1002/acm2.14158 37722769 PMC10860456

[33] Prentou G , Pappas EP , Prentou E , et al. Impact of systematic MLC positional uncertainties on the quality of single‐isocenter multi‐target VMAT‐SRS treatment plans. J Appl Clin Med Phys. 2022;23:e13708. doi:10.1002/acm2.13708 35733367 PMC9359048

[34] Middlebrook ND , Sutherland B , Kairn T . Optimization of the dosimetric leaf gap for use in planning VMAT treatments of spine SABR cases. J Appl Clin Med Phys. 2017;18(4):133‐139. doi:10.1002/acm2.12106 28574219 PMC5874863

[35] Glenn MC , Peterson CB , Followill DS , Howell RM , Pollard‐Larkin JM , Kry SF . Reference dataset of users’ photon beam modeling parameters for the eclipse, pinnacle, and raystation treatment planning systems. Med Phys. 2020;47:282‐288. doi:10.1002/mp.13892 31667870 PMC6980266

[36] Saez J , Hernandez V , Goossens J , De Kerf G , Verellen D . A novel procedure for determining the optimal MLC configuration parameters in treatment planning systems based on measurements with a Farmer chamber. Phys Med Biol. 2020;65(15):155006. doi:10.1088/1361-6560/ab8cd5 32330917

[37] Lin CY , Shiau AC , Ji JH , et al. A simple method for determining dosimetric leaf gap with cross‐field dose width for rounded leaf‐end multileaf collimator systems. Radiat Oncol. 2018;13(1):222. doi:10.1186/s13014-018-1164-1. Published 2018 Nov 13. 30424789 PMC6234646

[38] Xue X , Luan S , Ding Y , et al. Treatment plan complexity quantification for predicting gamma passing rates in patient‐specific quality assurance for stereotactic volumetric modulated arc therapy. J Appl Clin Med Phys. 2024;25:e14432. doi:10.1002/acm2.14432 38889335 PMC11492345

[39] Gardner SJ , Lu S , Liu C , Wen N , Chetty IJ . Tuning of Acuros XB source size setting for small intracranial targets. J Appl Clin Med Phys. 2017;18:170‐181. doi:10.1002/acm2.12091 PMC5689841 28470819

[40] Van Esch A , Kulmala A , Rochford R , Kauppinen J , Harju A . Testing of an enhanced leaf model for improved dose calculation in a commercial treatment planning system. Med Phys. 2022;49(12):7754‐7765. doi:10.1002/mp.16019 36190516

