# Supplementary material for: A novel protocol for parametrization of a beam model for small stereotactic beams
Source: J Appl Clin Med Phys. 2025 Jul 14;26(7):e70158. doi: 10.1002/acm2.70158 (PMC12257346; doi:10.1002/acm2.70158)
Supplement: Supplementary file 1 — Supporting Information [file ACM2-26-e70158-s001.docx]

| Source of uncertainty | Estimated uncertainty (%) | Contributions |
| --- | --- | --- |
| Measurement | ± 1-2 % | Jaw, MLC & detector positioning errors, Isocenter misalignments, Volume averaging |
| Spatial registration | ± 1 mm (±0.1-0.5 %) | Extrapolation of profiles from the TPS, Similarity metric, Tolerance… |
| Beam modelling | ± 2-3 % | AAA & Acuros modelling uncertainties, Energy fluence parametrizations… |
| Parameter optimization | ± 0.5-1 % | Field size weighting, depth averaging… |
| Data processing | ± 0.5-1 % | Interpolation, Resampling, discretization… |
